# Firearm Violence and Health in Policymaker Discourse: Mixed Methods Social Media Analysis

**DOI:** 10.2196/80397

**Published:** 2025-12-16

**Authors:** Vivek A Ashok, William J K Vervilles, Katherine S Kellom, Anyun Chatterjee, Isabella Ntigbu, Okechi Boms, Andrea Szabo, Joel A Fein, Therese S Richmond, Jonathan Purtle, Matthew D Kearney, Zachary F Meisel

**Affiliations:** 1 Division of General and Community Pediatrics, Cincinnati Children's Hospital Medical Center, 3333 Burnet Ave, Cincinnati, OH, 42229-3026, United States, 1 513-636-7106; 2 PolicyLab, Children's Hospital of Philadelphia, Philadelphia, PA, United States; 3 The Center for Violence Prevention, Children's Hospital of Philadelphia, Philadelphia, PA, United States; 4 Department of Pediatrics, School of Medicine, University of Cincinnati, Cincinnati, OH, United States; 5 Combined Internal Medicine-Pediatrics Residency Program, Department of Medicine, University of Pennsylvania, Philadelphia, PA, United States; 6 Qualitative Research Core, Children's Hospital of Philadelphia, Philadelphia, PA, United States; 7 Department of Pediatrics, Perelman School of Medicine, University of Pennsylvania, Philadelphia, PA, United States; 8 Emergency Medicine Residency Program, School of Medicine, Brown University, Providence, RI, United States; 9 Division of Emergency Medicine, Department of Pediatrics, Children's Hospital of Philadelphia, Philadelphia, PA, United States; 10 Leonard Davis Institute of Health Economics, University of Pennsylvania, Philadelphia, PA, United States; 11 Department of Biobehavioral Health Sciences, School of Nursing, University of Pennsylvania, Philadelphia, PA, United States; 12 Penn Injury Science Center, University of Pennsylvania, Philadelphia, PA, United States; 13 Department of Public Health Policy and Management, School of Global Public Health, New York University, New York, NY, United States; 14 Department of Family Medicine and Community Health, Perelman School of Medicine, University of Pennsylvania, Philadelphia, PA, United States; 15 Department of Emergency Medicine, Perelman School of Medicine, University of Pennsylvania, Philadelphia, PA, United States

**Keywords:** firearms, violence, social media, communication, health policy, prevention

## Abstract

**Background:**

Since 2019, firearm violence has remained the leading cause of death for US children and adolescents aged 1‐19 years. This crisis has spurred action from policymakers, health professionals, and advocates. However, political polarization has contributed to divergent views on the causes and appropriate responses to firearm violence. Communication by elected officials, especially on social media, plays a critical role in shaping public opinion and policy agendas. Understanding how state policymakers discuss firearm violence, including the use of causal blame, calls to action, and health-related narratives, can inform more effective public health strategies.

**Objective:**

This study aimed to examine how Pennsylvania state legislators discuss firearms and firearm violence on social media and assess the extent to which their messaging aligns with public health perspectives.

**Methods:**

We conducted a 2-phase mixed methods analysis of X (formerly known as Twitter; X Corp) posts by Pennsylvania state legislators from May 27, 2017, to July 26, 2022. Posts were grouped into 3 time periods surrounding the Tree of Life Synagogue mass shooting in Pittsburgh. Using a Boolean search strategy, we identified 4573 posts related to firearms and firearm violence. After removing reposts and non-English content, we randomly sampled 1491 (32.6%) original posts authored by 152 unique legislators. Posts were coded using a structured codebook based on the Multiple Streams Framework to capture rhetorical framing, causal blame, and policy content. Interrater reliability was high (Holsti coefficient >0.8). We used chi-square tests and multivariable logistic regression to assess associations between rhetorical elements and policy mentions, adjusting for time period.

**Results:**

Mass shootings were the most frequently referenced category of firearm violence, peaking after the Tree of Life shooting (22/43, 51% vs 91/118, 77.1% vs 140/220, 63.6%; *P*=.004), while firearm suicide was rarely discussed. Posts using advocacy frames were nearly 5 times more likely to mention policy (adjusted odds ratio [aOR] 4.67, 95% CI 3.55‐6.16), whereas those referencing mass shootings (aOR 0.54, 95% CI 0.37‐0.77) or emotional appeals (aOR 0.53, 95% CI 0.40‐0.69) were significantly less likely to do so. Most posts used general advocacy (aOR 2.97, 95% CI 2.13-4.13) and vague blame (aOR 8.26, 95% CI 6.02‐11.35), resulting in nonspecific policy suggestions. Posts that attributed blame to firearm access were strongly associated with specific policy proposals (aOR 6.37, 95% CI 4.29-9.47) and inversely associated with general policy mentions (aOR 0.26, 95% CI 0.17‐0.42). Only 9.4% (133/1422) of posts used health frames; when present, they more often referenced physical consequences (58/133, 43.6% vs 216/1358, 15.9%; *P*<.001).

**Conclusions:**

Pennsylvania legislators primarily focused on mass shootings and relied on emotional or symbolic language without proposing specific policies. Health frames were rare and typically focused on consequences rather than prevention. Findings highlight an opportunity to support policymakers with health-informed messaging strategies to promote actionable firearm violence prevention policies, particularly those addressing prevention.

## Introduction

Firearm violence and its devastating physical, psychological, and economic consequences in the United States have galvanized policymakers, health and public health professionals, researchers, law enforcement, and the judicial system to enact policies aimed at stopping this epidemic [1-5]. However, despite growing attention, deeply entrenched political polarization surrounding firearms has led to divergent views on the causes and consequences of firearm violence and the policies proposed to address it [6,7]. These divisions persist amid a steady rise in firearm violence, now the leading cause of death among children and adolescents aged 1‐19 years [4]. Moreover, Non-Hispanic Black or African American adolescents and young adults experience significantly higher rates of interpersonal firearm violence, while American Indian and Alaskan Native youth experience disproportionately high rates of firearm suicide, reflecting broader societal inequities [3,8-11]. This burden is often overlooked in policy discourse and media coverage, especially on social media, where selective framing can skew public perception [12]. Nonetheless, social media remains a powerful force in influencing policymaking through effective health and public health communication [13].

Elected officials play a central role in shaping the policy environment and represent a critical upstream political determinant of health [13,14]. Increasingly, legislators and their staff have turned to social media as a tool for issue engagement and political action [15-19]. Understanding how policymakers perceive, define, and communicate about firearm violence is essential to informing effective and equitable policy solutions. Social media offers a unique window into policymaker perspectives and directly impacts health policy agenda setting that is increasingly used in policy research [13,20-26]. It allows unfiltered access to policymakers’ unscripted viewpoints and positions without the framing and gatekeeping of traditional media [13]. Specifically, social media can reveal what causes are emphasized, what solutions are proposed, how certain incidents elicit reactions, and how specific populations are represented. Through this medium, policymakers can elevate public understanding of firearm violence; however, they can also perpetuate misinformation and misleading narratives that undermine evidence-based prevention strategies [27,28].

Despite the growing role of social media in shaping public discourse and policy agendas, no prior study has systematically examined how policymakers characterize firearm violence online. Most legislative action around firearm regulation and violence prevention occurs at the state level [29]. Understanding how the content and framing of state-level policymaker messages have evolved over time, how rhetorical strategies, such as causal blame and calls to action, correspond with policy discussions, and the degree to which firearm violence is conceptualized through and aligns with a health or public health lens is necessary to address this epidemic. Addressing these gaps is critical for many reasons. The ways in which firearm violence is framed—whether as a health or public health issue, a criminal justice concern, or a partisan talking point—influence the development and uptake of policies [30-38]. Furthermore, biased or inaccurate representations can advance policies that are misguided, ineffective, or inequitable by further entrenching health inequities and promoting stigma and bias of certain groups [27,28]. To better understand how recent sociopolitical discourse may align or misalign with public health and health care realities, we analyzed the language used by Pennsylvania state legislators in their social media posts from 2017 through 2022.

## Methods

### Setting, Data Collection, and Sampling

We conducted a 2-phased mixed methods analysis of X (formerly known as Twitter) posts related to firearms and firearm violence by Pennsylvania state legislators. This involved a systematic qualitative coding process using an iteratively developed codebook, followed by quantitative content analysis to identify trends in message framing, associations, and policy engagement. In order to capture the breadth of our discussion, firearm violence was broadly defined to include firearm suicide, any interpersonal firearm injury, and unintentional firearm injury.

We focused our analysis geographically on the Commonwealth of Pennsylvania for its heterogeneity in political party affiliation, urban and rural representation, firearm-related cultural norms, and diverse demographic makeup. We focused on state legislators because state-level policymaking has the most direct influence on firearm-related legislation, funding, and implementation, and because state legislative discourse reflects the primary locus of policy debate in this domain. We collected posts from May 27, 2017, to July 26, 2022, selecting a time frame that allowed us to examine the potential influence of major local and global events. We analyzed three distinct 15-month periods: (1) prior to the Tree of Life Synagogue shooting in Pittsburgh (from May 27, 2017 to October 26, 2018), (2) following that shooting through the onset of the COVID-19 pandemic (from October 27, 2018 to February 26, 2020), and (3) during the pandemic (from February 27, 2020 to July 26, 2022). Based on prior literature, using a local mass shooting as a fulcrum for time-varying analysis allows us to capture periods when firearm violence garners different levels of attention [39]. Furthermore, the COVID-19 pandemic was marked by an overall rise in firearm violence and firearm ownership and offered a distinct contextual period independent of the mass shooting event [40,41].

Our unit of analysis was individual X posts. Data were sourced from Quorum (Quorum Analytics), a public affairs software program that tracks policymaker social media engagements; data are available from 2007 to present [42]. As is standard practice, the research team paid a fee to access the Quorum database. Quorum adheres to public data usage guidelines set by X. The database provides metadata on the source of each post, the policymaker’s political party affiliation, the policymaker’s represented district, and the date posted.

**Figure 1.**
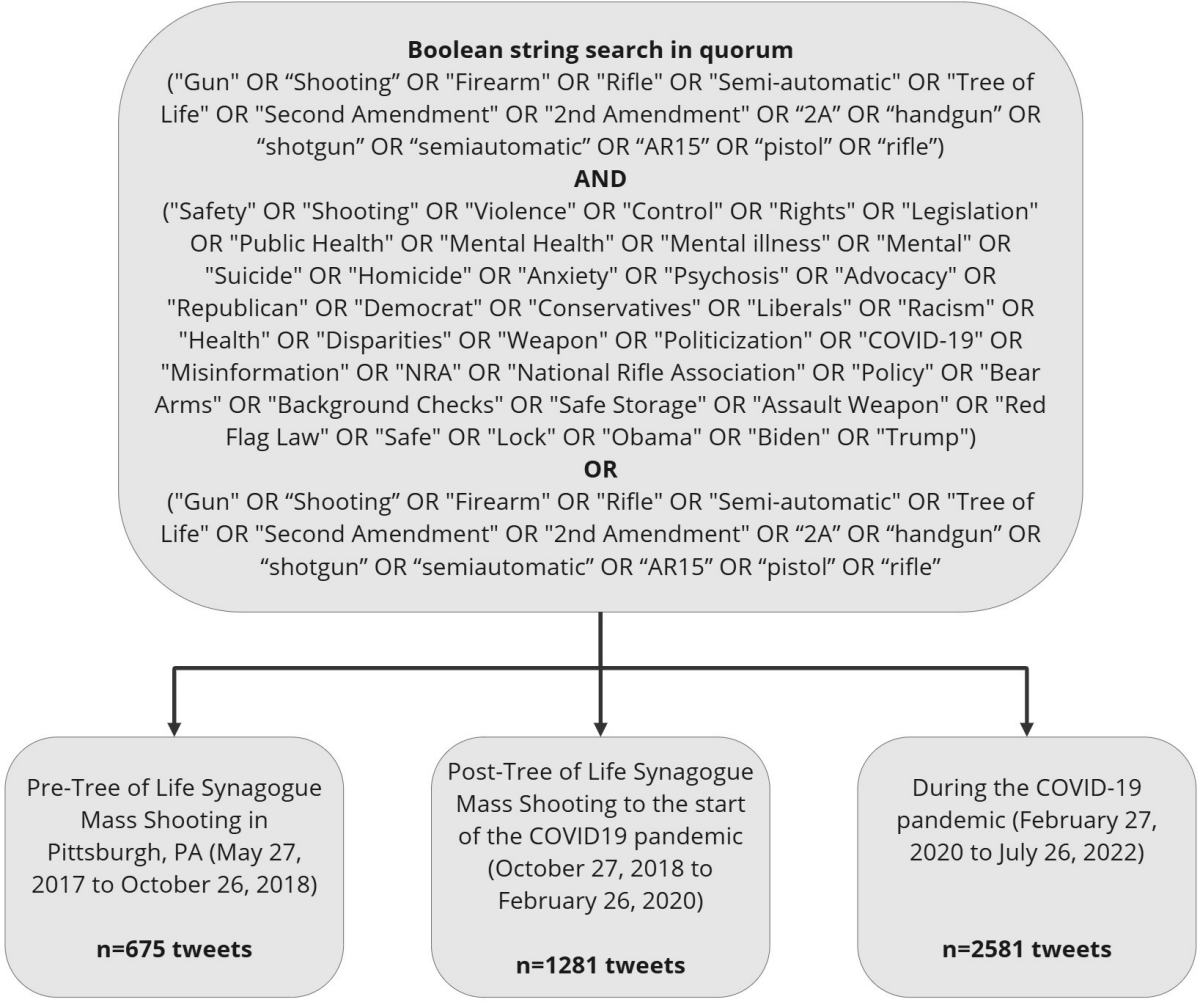
Identification of X posts by Pennsylvania state legislators describing firearms and firearm violence, 2017-2022.

Using Quorum’s advanced search features, we limited our query to Pennsylvania state legislators and the study period of interest. Pennsylvania state legislators created a total of 401,477 posts. Drawing on existing literature and in consultation with academic librarians, we created a Boolean string to capture posts relevant to discourse around firearms and firearm violence. For example, we included any post including the terms “gun(s),” “firearm(s),” “shooting(s),” AND “violence,” “safety,” etc (full list of search terms is provided in Figure 1) [43]. This search yielded 4573 eligible X posts, from which we excluded reposts and non-English posts. From the remaining pool, we drew a final simple random sample of 1491 (32.6%) posts authored by 152 unique users for in-depth coding and quantitative analysis. The final sample size was determined post hoc, once thematic saturation was achieved, defined as the point at which no new themes or codes emerged across successive batches of coded posts. This number also provided adequate representation across the study period to permit descriptive comparisons and multivariable logistic regression analyses. Each post was manually reviewed during coding to ensure relevance to the study topic.

### Qualitative Analysis

#### Codebook Development and Theoretical Framework

**Figure 2.**
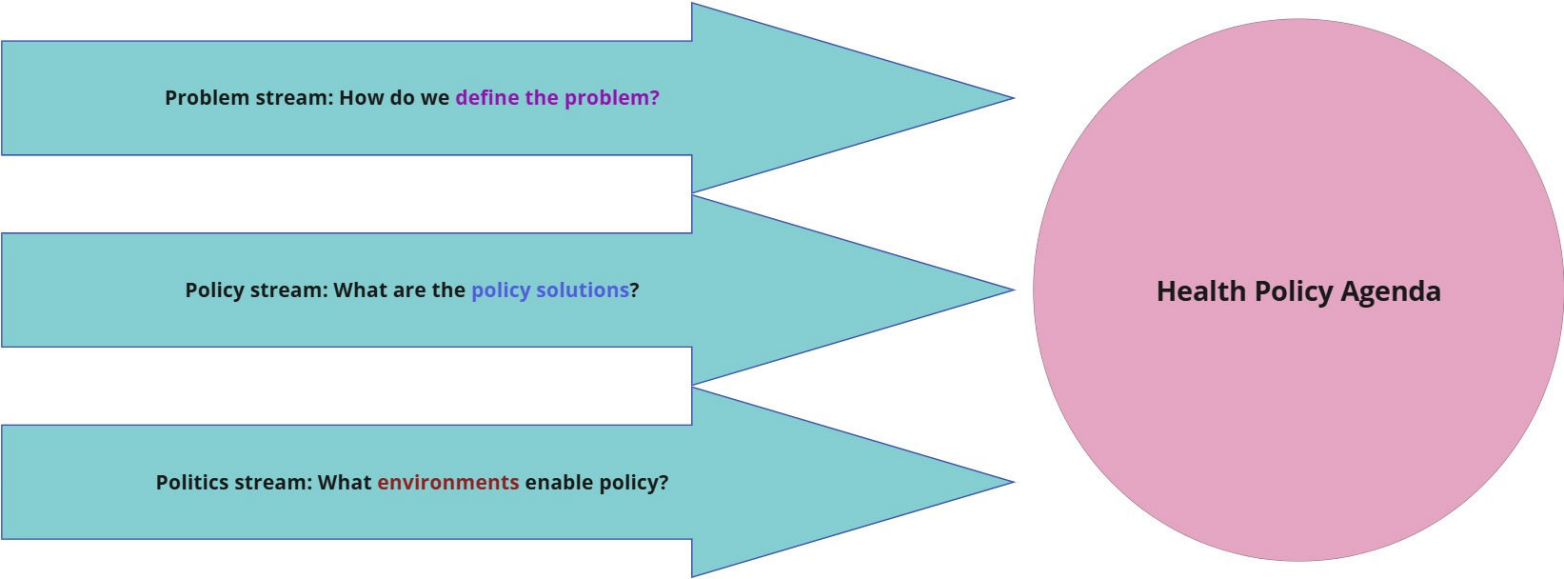
Kingdon’s multiple streams framework adapted for health policy discourse on firearm violence among Pennsylvania state legislators (2017-2022).

Five researchers (VAA, KSK, IN, MDK, and WJKV), with a range of expertise in firearm injury prevention research, health services research, social media, qualitative methods, health and medicine, and public health, collaboratively developed a 56-item codebook (Multimedia Appendix 1). Our coding framework was grounded in the Kingdon [44] Multiple Streams Framework (MSF) for Policy Agenda Setting (Figure 2 [45-47]). The MSF model has been used extensively in social and political sciences to evaluate policy agenda setting and offers an innovative way to conceptualize the pathway to health policy creation [47,48].

The MSF model describes 3 “streams” that influence policy agenda setting: the problem stream, policy stream, and politics stream. The problem stream refers to how a problem is defined and socially constructed. The policy stream relates to the development and advocacy of specific solutions. The politics stream refers to the sociopolitical forces that shape policy adoption. Our analysis focused on the problem and policy streams. We excluded the politics stream from our coding schema to maintain analytic focus on the content of the posts themselves and to minimize coder bias that could arise from subjective interpretations of external political context. However, recognizing that posts are not created in a vacuum and often reflect broader geopolitical events, we used the politics stream post hoc to contextualize our findings within relevant sociopolitical moments. Within the problem stream, we used the Policy Frames Codebook*,* created by Boydstun et al [49], to identify salient frames used to describe firearms and firearm violence (Table 1).

**Table 1.** Frames, definitions, and example X posts by Pennsylvania state legislators (2017-2022).

Frame	Definition	Example X post
Morality	Any perspective—or policy objective or action (including proposed action)—that is compelled by moral doctrine or interpretation, logic, duty, honor, honesty or integrity, righteousness or any other sense of ethics or social responsibility. A calculated way for someone to think about right and wrong rather than emotion. Often, the phrase “common sense” is used to connote logic.	“Thoughts and prayers do not protect our children. I will not participate in these actions when we can pass common sense gun laws TODAY.”
Advocacy or Endorsement	A way of presenting information that promotes a particular viewpoint, policy, or action. It is designed to persuade the audience to support a specific cause, agenda, or position. This type of frame often includes subjective elements, such as endorsements, recognition of efforts, calls to action, and expressions of support or opposition. This frame can be general or specific advocacy. For example, it can include advocating for general gun violence prevention (viewpoint) or protecting the rights of firearm owners through legislation (policy). It can also include advocating or promoting one’s own actions toward advancing a specific goal. A vote or sponsorship of a bill is an endorsement.	“We cannot wait until the next Columbine, or Sandy Hook, or Parkland, or Uvalde. We need to act now. Lives depend on us. Help send a message to the Majority leaders and urge them to advance gun-safety legislation”
Emotional Appeal or Moral Outrage	Uses words and phrases to evoke sympathy, sorrow, and a sense of tragedy. Uses phrases to express injustice and moral outrage, which can motivate audiences to act against an issue. Using facts or statistics to highlight the sheer scope of an issue and evoke a sense of moral imperative.	“Today is a day of mourning and reflection as we mark one year since the mass shooting at the Tree of Life building in Pittsburgh. Eleven were killed and six others injured in the deadliest attack on the Jewish community in the United States. Remember. Repair. Together.”
Cultural Identity	Relates to social norms, trends, values, and customs. For example, it can include hunting and sporting events and shooting season in Pennsylvania. It may also refer to tradition and firearms as heritage items.	“Rifle season starts Saturday and it’s important to remember to be safe while hunting or hiking in PA’s outdoors. Have fun and good luck to all the hunters out there!”
Equality or Equity	Equality or inequality with which laws, punishment, rewards, and resources are applied or distributed among individuals or groups. Also, the balance between the rights or interests of one individual or group compared to another individual or group.	“The signs and voices chanted about black lives and gun violence, about justice reform, about the systemic underfunding of schools in black and brown neighborhoods. about healthcare, housing, transportation, minimum wage. So much undone. (Yet to be done.)”
Criminology and Policing	Framing gun violence within the context of crime and law enforcement. Specifically, if the problem or solutions are grounded in crime prevention or law enforcement interventions (eg, longer sentences for perpetrators or criminal penalties for gun-related offenses); portrayals of gun violence that are intertwined with the criminal justice system (eg, highlighting gang violence and armed robberies); or involvement of law enforcement as primary responders (as opposed to health care workers or community members).	“Within Philadelphia, effective and non-lethal policing is also essential to healing communities that have been historically overpoliced and over incarcerated. It will take all stakeholders to end the scourge of gun violence! #EnoughIsEnough”
Constitutionality and Jurisprudence	The constraints imposed on or freedoms granted to individuals, government, and corporations via the Constitution, Bill of Rights, and other amendments, or judicial interpretation. This deals specifically with the authority of government to regulate and the authority of individuals or corporations to act independently of government. For example, civil libertarianism, citing encroachment on constitutional rights and individual liberties, most commonly the Second Amendment. Local laws or policies wrapped in legal or lawmaker language. Citations of legal cases are also included in this frame.	“I was proud to stand up for the Second Amendment rights of law-abiding Pennsylvanians today. The bills we approved will allow lawful gun owners to carry without a permit and maintain uniformity of state laws in every municipality in the state.”
Security, Defense, or Protection	Safety, threats to security, and protection of one’s person, family, in-group, nation, etc. Generally, it is an action or a call to action that can be taken to protect the welfare of a person, group, nation, sometimes from a not yet-manifested threat. In this frame, the coder asks if the post is using rhetoric related to security, defense, or protection to persuade you to take a stance or delineates specific steps to provide that security, defense, or protection. For example, this frame could include lauding the use of a firearm or gun for self-protection or protecting the community, or protecting the community from gun violence itself.	“I’ve introduced legislation to ensure that we are doing everything we can to protect the residents of the commonwealth. I will continue to be an advocate for legislative initiatives that respect the Second Amendment while promoting gun safety.”
Public Opinion	References to general social attitudes, polling, and demographic information, as well as implied or actual consequences of diverging from or “getting ahead of” public opinion or polls. Often uses the phrase “most people support.”	“People demand that we act & pass common sense gun violence prevention bills like ERPO.”
Health and Public Health	Is the public health approach to curb gun violence, as described by the Centers for Disease Control and Prevention (CDC), mentioned (must meet one criterion): (1) Does the post cite statistics or the importance of research, generating data, risk factors, etc? (2) Does the post mention using evidence-based practices or science? (3) Does the post mention evaluating the implementation of the methods used to prevent firearm violence?	Example “Did you know? Studies suggest that waiting period law may reduce firearms suicide rates by 7% to 11%. That’s around 2500 lives a year.”
Partisan	Defines the problems or solutions of gun violence through a partisan lens by touting one political party over another or castigating a specific political party.	“This is absolutely ridiculous. We are talking about thousands of lives lost due to gun violence. We will not be silenced. We will keep fighting for what’s right! Senate Republicans are now trying to silence [deidentified]. This is a typical move the GOP makes when they are clearly losing the argument.”
Informational	The entire post should convey information, which could include statistics, public health recommendations, and information about an event, campaign, or vigil. While neutral on the surface, it may not be felt as neutral since authors tend to use jarring statistics to drive home a point. There should be no partisan language in a post that is information-framed.	“SB565 reduces the age a person can carry a firearm from 21 to 18 and does not require a license to openly carry firearms in cities like Philadelphia.”
Religiosity or Spirituality	Relates to tying the right to bear a firearm or firearm violence to religiosity or spirituality. For example, “God given right” or “may God bless this state”	“Practically every gun tragedy is a preventable tragedy, if you don’t believe the myth that our Creator intended us to have the God-like ability to destroy human life”

The codebook was developed iteratively through group discussion and consensus. Guided by the MSF, the research team deductively coded posts using these predefined frames and inductively refined the codebook through group discussion and consensus. New or modified frames (eg, health and public health, criminology and policing, and advocacy or endorsement) were added as themes emerged during pilot coding. We coded a subset of 6.7% (100/1491) of the total analytic sample to refine and validate our codebook. We reviewed the data to ensure that key concepts within the problem and policy streams were sufficiently represented in the sample and well captured by our codebook. Throughout this process, discrepancies in interpretation were resolved with team-based consensus and subsequent revisions.

#### Coding Process

A final sample of 1491 posts was coded using a structured instrument developed in REDCap (Research Electronic Data Capture; Vanderbilt University). Seven coders (VAA, IN, WJKV, OB, AS, KSK, and AC) participated in this process, with VA serving as the reference coder. To assess interrater reliability, 10.6% (159/1491) posts of the final sample were coded by at least 2 team members. We calculated the Holsti coefficient for agreement, achieving average coefficients greater than 0.8 across domains. Consistent with qualitative coding conventions for larger teams, 1 coder (VA) served as the dominant coder, and all other coders were trained to achieve a Holsti coefficient >0.8 relative to VA. Each coding pair (VA and a secondary coder) independently coded a subset of 10‐20 posts, reviewed discrepancies, and iterated until consensus and the reliability threshold were achieved. Double-coded subsets were retrospectively amended to reflect consensus codes. To prevent coder drift, all coders participated in one to two double-coding sessions for every 250‐500 posts coded.

To minimize potential coder bias, all coders were blinded to the identity and political affiliation of the poster. In addition, embedded hyperlinks were not coded, as these sometimes revealed the identity or political affiliation of the author.

#### Measures and Statistical Analysis

Coded variables were applied at the post level, classified as binary or categorical, and entered into REDCap. Within the problem stream, we measured whether posts referenced general descriptors of violence, including the type of incident, location, and characteristics of violence survivor or perpetrators if a specific interpersonal firearm violence incident was discussed. These characteristics are conceptualized as part of the problem definition. Mentions of violence survivor or perpetrator identity were categorized by factors, if known or shared, such as age, race and ethnicity, gender identity or sexual orientation, mental health status, and involvement with the criminal justice system. Posts expressing symbolic responses, such as “thoughts and prayers,” solidarity, or remembrance, were also coded. We identified calls to action, including general appeals for change, specific policy recommendations, or encouragement to vote. Thirteen rhetorical frames were coded, encompassing moral arguments, advocacy language, emotional appeals, criminology and policing narratives, public health framing, cultural identity, partisanship, and equity. To capture all post elements, codes were nonmutually exclusive and could be used in combination with each other.

Within the policy stream, we coded whether posts assigned causal blame (ie, attribution of responsibility for a given action to specific individuals, groups, or systems) to any of 17 distinct sources, such as mental health, access to firearms, weak laws, racism, political parties, the firearm lobby, substance use, or social media. We defined causal blame as statements assigning responsibility for firearm violence to specific actors or systems (eg, weak laws and the firearm lobby), whereas rhetorical frames referred to the broader narrative lens through which the issue was discussed (eg, advocacy, emotion, and partisanship). These are conceptually and analytically distinct. We also coded references to the consequences of firearm violence, including mental and physical health impacts, economic costs, the cycle of violence, secondary trauma, and harm to community well-being. Finally, we assessed whether posts endorsed or proposed specific policies, such as (1) restrictive or permissive firearm ownership laws, (2) support or funding for community violence intervention programs or law enforcement, (3) initiatives targeting the firearm industry, and (4) policies addressing mental health or substance use.

We merged our coded dataset with the original Quorum dataset using unique identifiers to retain metadata on post date and author. We calculated the proportion of X posts that referenced each code and used chi-square tests to assess whether these proportions varied significantly across the three 15-month time frames. We applied Bonferroni correction for multiple comparisons with a significance level of P=0.02 when appropriate. All statistical analysis was performed using STATA 18.0 (StataCorp LLC) software.

We conducted multivariable logistic regression, adjusting for time period, to examine the associations between policy mentions and rhetorical elements, such as calls to action, types of shootings, causal blame attributions, and thematic frames. While rhetorical frames, causal blame attributions, and types of firearm violence may co-occur in individual posts, they were coded as distinct constructs and modeled as independent predictors in our regression analyses. Our primary model examined predictors of any policy-related content being mentioned, using variables selected based on descriptive prevalence and refined through significance testing and model fit. Then, we ran a series of additional models to assess whether specific categories of violence, causal blame attributions, or frames were individually associated with policy-related content.

To further explore the relationship between rhetorical framing and policy specificity, we stratified our policy outcome variable to distinguish between general (nonspecific) and specific policy proposals and re-estimated our model to examine predictors of each category. Predictors included categories of firearm violence mentioned, types of call to action, framing devices, and causal blame attributions. Finally, we conducted a subgroup analysis comparing X posts that did and did not use health frames, using chi-square tests to identify which topics were more likely to be mentioned when firearm violence was conceptualized as a health issue. Adjusted odds ratios (aORs) with 95% CIs were reported, using robust SEs.

### Ethical Considerations

The dataset contained only publicly available information on X from the offices of elected officials, and therefore did not meet the criteria for human subjects research. All posts, data, and user IDs were deidentified for this study.

## Results

### Descriptive Results

We analyzed 1491 unique X posts by Pennsylvania legislators related to firearms and firearm violence. Of these, 210 (14%) were posted 15 months prior to the Tree of Life Synagogue shooting, 415 (27.8%) in the 15 months following the shooting through the onset of the COVID-19 pandemic, and 866 (58%) during the first 15 months of the pandemic. We present results using the MSF, starting with the problem stream (Table 2) and the policy stream (Table 3).

**Table 2.** The problem stream–unadjusted comparisons of specific topic mentions on X (formerly known as Twitter) by Pennsylvania state legislators about firearms and firearm violence during 3 study time periods (2017-2022).

Topic	Time period 1 (n=210), n (%)	Time period 2 (n=415), n (%)	Time period 3 (n=866), n (%)	Totals (n=1491), n (%)	*P* values
Descriptions of firearm violence					
Mentions of specific type of firearm violence	43 (20.5)	118 (28.4)	220 (25.4)	381 (25.6)	.10
Mass shooting^a^^,^^b^	22 (51.2)	91 (77.1)	140 (63.6)	253 (66.4)	.004
Suicide or intentional self- inflicted harm	3 (7)	11 (9.3)	18 (8.2)	32 (8.4)	.88
Unintentional or accidental injury	1 (2.3)	2 (1.7)	8 (3.6)	11 (2.9)	.58
Interpersonal or community violence^b^	11 (25.6)	13 (11)	55 (25)	79 (20.7)	.007
Other type of shooting	6 (14)	4 (3.4)	22 (10)	32 (8.4)	.04
Mentions of specific locations of shooting	48 (22.9)	107 (25.8)	204 (23.6)	358 (24.1)	.62
School or school grounds	11 (22.9)	12 (11.2)	39 (19.1)	62 (17.3)	.12
Home	1 (2.1)	0 (0)	3 (1.5)	4 (1.1)	.40
Place of worship^a^^,^^b^^,^^c^	1 (2.1)	52 (48.6)	38 (18.6)	91 (25.4)	<.001
Neighborhood or community	3 (6.2)	5 (4.7)	14 (6.9)	22 (6.1)	.75
Place of business	3 (6.2)	5 (4.7)	17 (8.3)	25 (7)	.47
City or county^b^	28 (58.3)	47 (43.9)	120 (58.8)	195 (54.5)	.04
Other location	2 (4.2)	0 (0)	4 (2)	6 (1.7)	.15
Mentions of a vigil, remembrance event, or campaign (such as #WearOrange)	14 (7.6)	47 (12.6)	77 (10.1)	138 (9.3)	.17
Mentions of relationship of shooter to violence survivor	5 (2.4)	6 (1.4)	34 (3.9)	45 (3)	.04
Intimate partner^b^^,^^c^	4 (80)	3 (50)	1 (2.9)	8 (17.8)	<.001
Parent or guardian	0 (0)	0 (0)	0 (0)	0 (0)	—^d^
Family member	0 (0)	0 (0)	1 (2.9)	1 (2.2)	.85
Law enforcement	1 (20)	1 (16.7)	19 (55.9)	21 (46.7)	.09
Other authority figure	0 (0)	0 (0)	1 (2.9)	1 (2.2)	.85
Unknown to violence survivor	0 (0)	1 (16.7)	5 (14.7)	6 (13.3)	.64
Other relationship	0 (0)	1 (16.7)	9 (26.5)	10 (22.2)	.40
Expressions of symbolic responses	34 (16.2)	91 (21.9)	169 (19.5)	294 (19.7)	.23
“Thoughts and Prayers”^a^	17 (50)	22 (24.2)	51 (30)	90 (30.6)	.02
Solidarity^b^	11 (32.4)	26 (28.6)	76 (45)	113 (38.4)	.03
Remembrance^c^	7 (20.6)	58 (63.7)	68 (40.2)	133 (45.2)	<.001
Mentions of calls to action	61 (29)	103 (24.8)	293 (33.8)	457 (30.7)	.004
General call to action	46 (75.4)	80 (77.7)	205 (70)	331 (72.4)	.28
Specific call to action	31 (51.7)	43 (41.7)	130 (44.5)	204 (44.8)	.46
Calls to vote^c^	9 (29)	5 (12.2)	14 (10.9)	28 (13.9)	.03
Issue framing	199 (94.8)	390 (94)	833 (96.2)	1422 (95.4)	.35
Morality	10 (5)	26 (6.7)	50 (6)	86 (6)	.73
Advocacy or endorsement	135 (67.8)	283 (72.6)	633 (76)	1051 (73.9)	.049
Emotional appeal or moral outrage^c^	76 (38.2)	171 (43.8)	412 (49.5)	659 (46.3)	.008
Cultural identity	3 (1.5)	13 (3.3)	27 (3.2)	43 (3)	.40
Equality or equity	4 (2)	12 (3.1)	26 (3.1)	42 (3)	.70
Criminology and policing	8 (4)	16 (4.1)	46 (5.5)	70 (4.9)	.46
Constitutionality and jurisprudence	14 (7)	32 (8.2)	75 (9)	121 (8.5)	.65
Security, defense, or protection	24 (12.1)	41 (10.5)	81 (9.7)	146 (10.3)	.61
Public opinion	7 (3.5)	13 (3.3)	38 (4.6)	58 (4.1)	.55
Health and public health^b^	20 (10.1)	50 (12.8)	63 (7.6)	133 (9.4)	.01
Partisan^b^^,^^c^	17 (8.5)	28 (7.2)	181 (21.7)	226 (15.9)	<.001
Informational^c^	35 (17.6)	50 (12.8)	84 (10.1)	169 (11.9)	.01
Religiosity or spirituality	4 (2)	10 (2.6)	14 (1.7)	28 (2)	.55

a Post hoc pairwise comparisons significant for Time 1 versus Time 2.

b Post hoc pairwise comparisons significant for Time 2 versus Time 3.

c Post hoc pairwise comparisons significant for Time 1 versus Time 3.

d Not available.

**Table 3.** The policy stream-unadjusted comparisons of specific topic mentions on X (formerly known as Twitter) by Pennsylvania state legislators about firearms and firearm violence during 3 study time periods (2017-2022).

Topic	Time period 1 (n=210), n (%)	Time period 2 (n=415), n (%)	Time period 3 (n=866), n (%)	Totals (n=1491), n (%)	*P* values
Causal blame or causal attributions	100 (47.6)	180 (43.4)	487 (56.2)	767 (51.4)	<.001
Mental health or illness	2 (2)	7 (3.9)	22 (4.5)	31 (4)	.50
Weak firearm laws	40 (40)	79 (43.9)	199 (40.9)	318 (41.5)	.74
Increased ownership, access, or availability of firearms	16 (16)	27 (15)	106 (21.8)	149 (19.4)	.10
Decreased possession of firearms	0 (0)	0 (0)	5 (1)	5 (0.7)	.24
Sociopolitical and structural determinants of health	0 (0)	2 (1.1)	12 (2.5)	14 (1.8)	.20
Stereotyped, Biased, or Racist beliefs of a group of persons^a^^,^^b^	0 (0)	10 (5.6)	34 (7)	44 (5.7)	.02
Guns in the hands of wrong people	9 (9)	13 (7.2)	19 (3.9)	41 (5.3)	.05
Gang Violence or Organized crime	0 (0)	0 (0)	1 (0.2)	1 (0.1)	.75
Social media	0 (0)	0 (0)	1 (0.2)	1 (0.1)	.75
Individuals or groups of policymakers (eg, Philadelphia Attorney General)	15 (15)	20 (11.1)	83 (17)	118 (15.4)	.17
Causal blame attributed to the violence survivor	0 (0)	0 (0)	1 (0.2)	1 (0.1)	.75
Substance use	0 (0)	0 (0)	0 (0)	0 (0)	—^c^
Firearm industry and commercial practices	0 (0)	2 (1.1)	8 (1.6)	10 (1.3)	.41
US firearm culture	2 (2)	2 (1.1)	6 (1.2)	10 (1.3)	.80
The firearm lobby^b^	13 (13)	9 (5)	14 (2.9)	36 (4.7)	<.001
A specific political party or affiliation^d^^,^^b^	8 (8)	7 (3.9)	131 (26.9)	146 (19)	<.001
Hate or hate crimes^a^	0 (0)	13 (7.2)	26 (5.3)	39 (5.1)	.03
Other causal blame category	27 (27)	40 (22.2)	86 (17.7)	153 (19.9)	.07
Consequences	44 (21)	129 (31.1)	298 (34.4)	471 (31.6)	<.001
Economic or Financial	0 (0)	2 (1.6)	5 (1.7)	7 (1.5)	.69
Physical harm or disability	25 (56.8)	78 (60.5)	171 (57.4)	274 (58.2)	.82
Mental health or illness	1 (2.3)	4 (3.1)	12 (4)	17 (3.6)	.79
Furthering the cycle of violence	10 (22.7)	23 (17.8)	73 (24.5)	106 (22.5)	.32
Secondary trauma to cosurvivors of violence	12 (27.3)	32 (24.8)	79 (26.5)	123 (26.1)	.92
General individual or community well-being^a^	10 (22.7)	11 (8.5)	50 (16.8)	71 (15.1)	.03
Other consequence	0 (0)	3 (2.3)	7 (2.3)	10 (2.1)	.59
Policies mentioned	102 (48.6)	196 (47.2)	494 (57)	792 (53.1)	.002
Generic of nonspecific policies (reduction programs, initiatives, or reforms)	68 (66.7)	120 (61.2)	288 (58.3)	476 (60.1)	.27
Restrictive firearm policies	21 (20.6)	55 (28.1)	115 (23.3)	191 (24.1)	.28
Permissive firearm policies^d^	7 (6.9)	6 (3.1)	55 (11.1)	68 (8.6)	.002
Funding for CVIP/HVIP programs^d^^,^^b^^,^^e^	2 (2)	5 (2.6)	45 (9.1)	52 (6.6)	<.001
Funding for firearm research	1 (1)	0 (0)	4 (0.8)	5 (0.6)	.43
Address mental health and illness	1 (1)	4 (2)	12 (2.4)	17 (2.1)	.65
Address substance use disorder	0 (0)	0 (0)	2 (0.4)	2 (0.3)	.55
Funding or support for law enforcement^a^	3 (2.9)	0 (0)	8 (1.6)	11 (1.4)	.09
Hold firearm industry accountable	1 (1)	1 (0.5)	3 (0.6)	5 (0.6)	.88
Hold the gun lobby accountable	2 (2)	3 (1.5)	1 (0.2)	6 (0.8)	.06
Address structural determinants of violence	0 (0)	1 (0.5)	7 (1.4)	8 (1)	.31
Other policy mentioned	5 (4.9)	16 (8.2)	28 (5.7)	49 (6.2)	.40

a Post hoc pairwise comparisons significant for Time 1 versus Time 2.

b Post hoc pairwise comparisons significant for Time 1 versus Time 3.

c Not applicable.

d Post-hoc pairwise comparisons significant for Time 2 versus Time 3.

e CVIP: Community-Based Violence Intervention Program/HVIP: Hospital-Based Violence Intervention Program.

Among 381 posts that mentioned a specific category of firearm violence, mass shootings were the most common (253, 66.4%), with statistically significant variation across the 3 time periods (22/43, 51% vs 91/118, 77.1% vs 140/220, 63.6%; *P*=.004). Interpersonal or community violence was the next most frequently mentioned category (79, 20.7%), with a significant increase after the onset of the pandemic. Suicide and unintentional injury were rarely discussed.

When specific locations were mentioned (358 posts), legislators most often named cities or counties (195/358, 54.5%) and places of worship (91/358, 25.4%). Mentions of places of worship peaked following the Tree of Life Synagogue shooting. Additionally, a significant portion of posts (138/1491, 9.3%) described vigils or remembrance events, aligning with the symbolic frame of “Remembrance,” which was the most commonly expressed symbolic response (133/294, 45.2% posts) and increased significantly over time. “Solidarity” and “Thoughts and Prayers” were also common expressions (113/294, 38.4% and 90/294, 30.6%) without significant changes over time. Pennsylvania legislators also mentioned “Calls to Action” in 457/1491 (30.7%) posts of the time, with the majority representing general calls to action (331/457, 72.4%) rather than specific ones (204/457, 14.2%).

The most common frames included advocacy or endorsement (1051/1422, 73.9% posts), emotional appeal or moral outrage (659/1422, 46.3%), partisan framing (226/1422, 15.9%), and informational framing (169/1422, 11.9%). Of these, partisan frames increased significantly by 14.5% (*P<*.001) during the COVID-19 pandemic, while emotional and informational frames differed significantly between the first and third time periods. Health or public health frames were rare, appearing in less than 1 in 10 (133/1422, 9.4%) posts. Pairwise comparisons between frames indicated that posts using advocacy or emotional frames were significantly less likely to co-occur with informational frames (*r*=–0.24; *P<*.001 and *r*=–0.18; *P<*.001, respectively). Religion and cultural frames were positively correlated (*r*=0.15; *P<*.001).

Among the 767/1491 (51.4%) posts that attributed causal blame, the most common attributions were to weak firearm laws—a nonspecific category where authors criticized laws as generally insufficient without referencing a particular statute or policy (318/767, 41.5% posts)—and to increased firearm ownership, access, or availability (149/767, 19.4% posts). Legislators also frequently blamed individual policymakers (118/767, 15.4%) or political parties (146/767, 19%), with blame attribution toward political parties significantly increasing by 23% during the COVID-19 pandemic period. A majority of posts (792/1491, 53.1%) included a mention of at least one nonspecific or specific policy. Nonspecific policy mentions (eg, “gun reform,” “initiatives,” or “common-sense laws”) were cited more often than specific policy proposals (476/792, 60.1%). Among posts describing policies, nearly a quarter (191/792, 24.1%) referenced policy proposals that restrict access to firearms, such as background checks or extreme risk protection orders.

### Analytic Results: Primary Model

**Figure 3.**
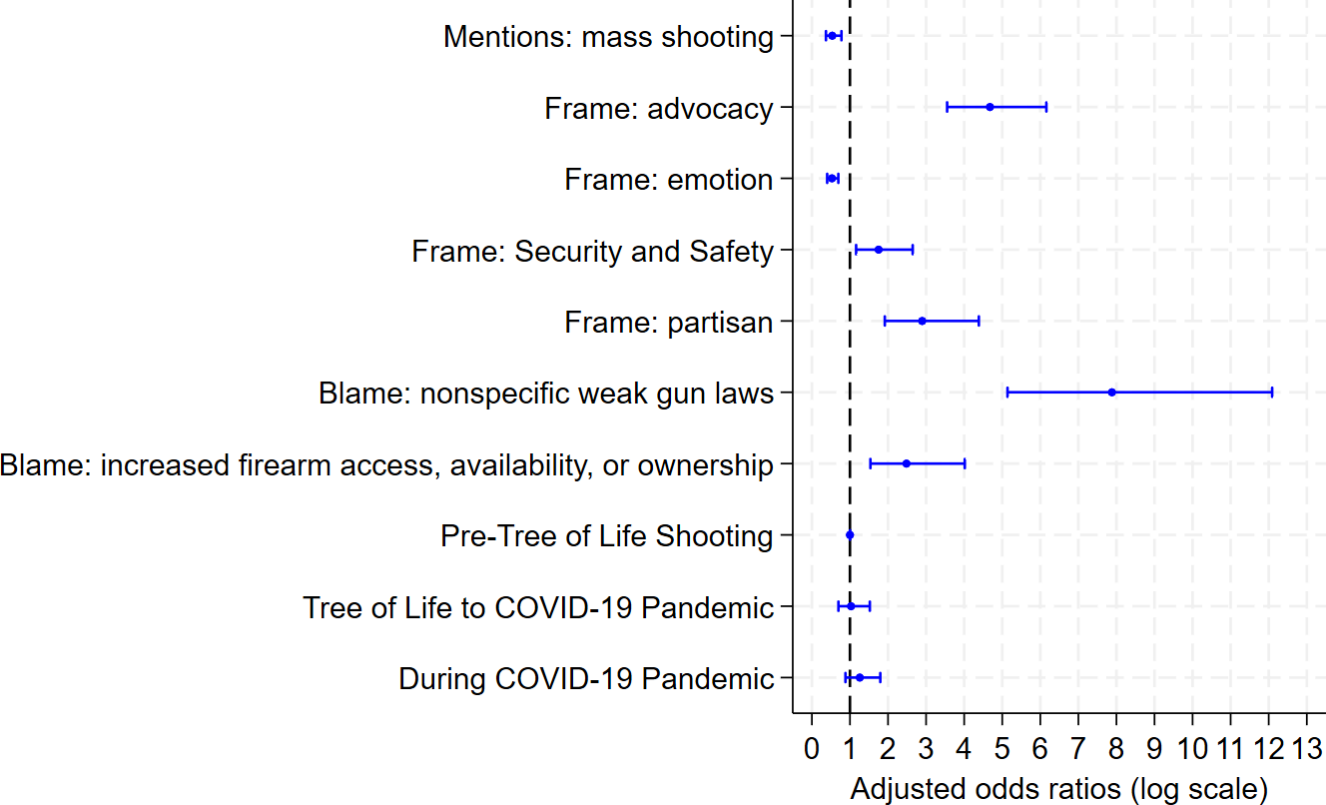
Adjusted odds ratios for predictors of any policy mention in X posts by Pennsylvania state legislators (2017-2022).

Adjusting for time period, our analysis of the relationship between any policy mention and rhetorical predictors derived from our descriptive content analysis revealed several features that were independently and significantly associated with policy-related content (Figure 3). Certain types of rhetorical framing and causal blame attributions were associated with policy mentions. For instance, posts that used advocacy frames had an almost 5 times greater odds of including any policy mention (aOR 4.67, 95% CI 3.55‐6.16) in addition to those that used partisan rhetorical framing (aOR 2.89, 95% CI 1.91‐4.38). Additionally, posts casting causal blame on weak firearm laws (aOR 7.88, 95% CI 5.14‐12.09) were significantly more likely to include any policy mention. In contrast, posts referencing mass shootings (aOR 0.54, 95% CI 0.37‐0.77) or using emotional frames (aOR 0.53, 95% CI 0.40‐0.69) were less likely to mention policy. The odds of any policy mention remained stable after holding time constant throughout all 3 time periods. Neither of the latter time periods was significantly associated with policy mentions, suggesting associations between any policy mention and rhetorical elements were consistent across time.

### Analytic Results: Stratified Policy Model

**Figure 4.**
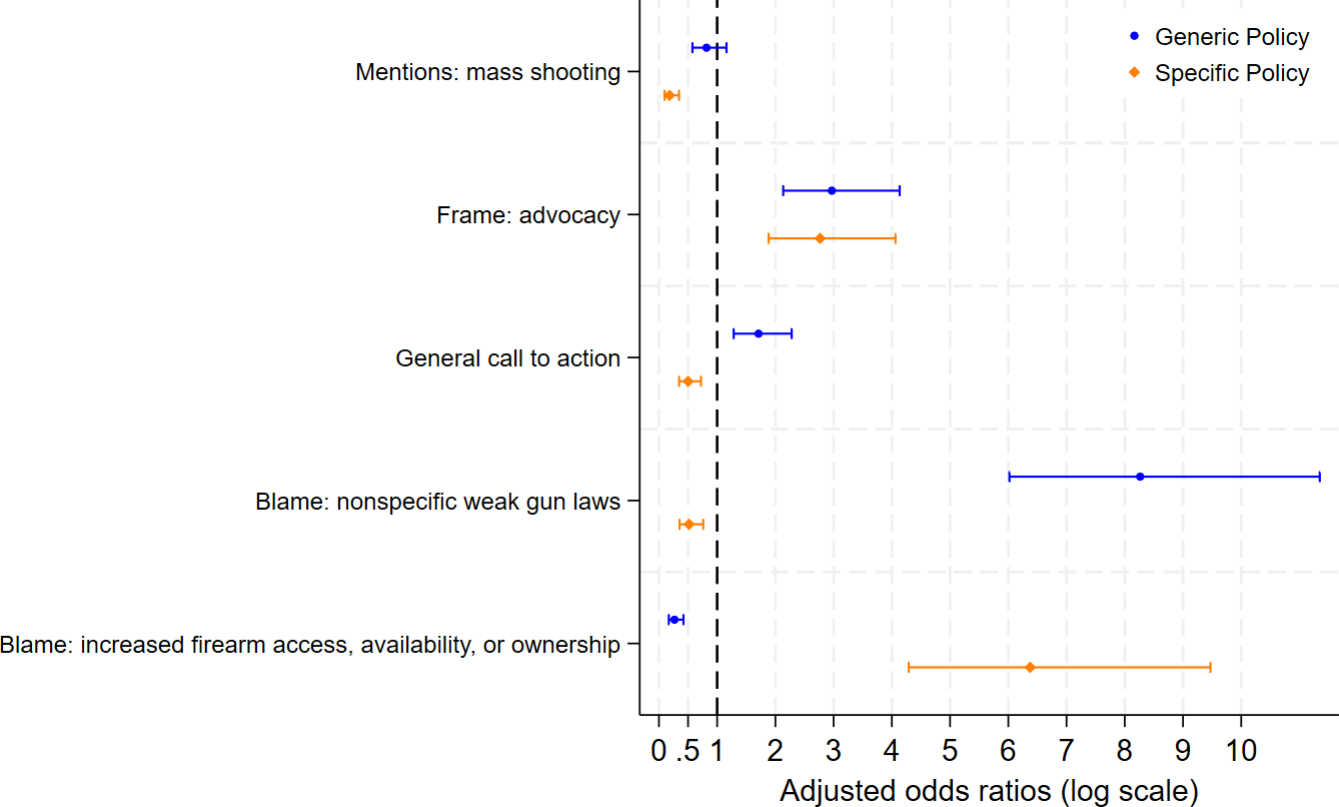
Adjusted odds ratios for predictors of generic versus specific policy mentions in X posts by Pennsylvania state legislators (2017-2022).

When stratifying by policy specificity, we observed notable differences (Figure 4). Posts using advocacy frames were significantly associated with both nonspecific (aOR 2.97, 95% CI 2.13‐4.13) and specific policy mentions (aOR 2.77, 95% CI 1.88‐4.06). Causal blame directed toward weak firearm laws independently predicted nonspecific policy mentions (aOR 8.26, 95% CI 6.02‐11.35) but was inversely associated with specific policy mentions (aOR 0.50, 95% CI 0.35‐0.72). In contrast, posts specifying blame toward increased access, availability, or ownership of firearms were more likely to reference specific policies (aOR 6.37, 95% CI 4.29‐9.47) and were negatively associated with nonspecific policy mentions (aOR 0.26, 95% CI 0.17‐0.42). General calls to action were also associated with increased odds of nonspecific policy mentions (aOR 1.71, 95% CI 1.28‐2.27) and decreased odds of specific policy mentions (aOR 0.50, 95% CI 0.35‐0.72). Finally, posts referencing mass shootings were the least likely to mention specific policies (aOR 0.18, 95% CI 0.10‐0.35).

### Individual Causal Blame, Shooting Type, and Frame Models

Three additional models, adjusted for time period, identified independent relationships between any policy mention and type of shooting, causal blame attributions, and rhetorical framing.

In the model assessing the relationship between shooting type, posts referencing mass shootings had a significantly lower odds of any policy mention (aOR 0.35, 95% CI 0.26‐0.47), while posts about firearm suicide had an almost 3 times greater odds of including a policy mention (aOR 2.81, 95% CI 1.18‐6.70). Other categories of firearm violence were not significantly associated with policy mentions. Posts made during the COVID-19 period had higher odds of including a policy mention compared to those posted prior to the Tree of Life shooting (aOR 1.51, 95% CI 1.11‐2.05; *P*=.009).

In the causal blame model, posts that attributed blame to increased firearm availability, accessibility, and ownership (aOR 3.26, 95% CI 1.98‐5.37) or to a specific political party (2.71, 95% CI 1.66‐4.43) were significantly associated with any policy mention. Although only 4% (31/767) of posts attributed firearm violence to mental health, these posts had significantly higher odds of any policy mention (aOR 4.60, 95% CI 1.55‐13.61). Other causal blame attributions were not associated with mentions of policy. The time period was not significantly associated with the likelihood of policy mention.

Finally, in the rhetorical frame model, posts using advocacy or endorsement (aOR 6.19, 95% CI 4.69‐8.16), public opinion (aOR 2.41, 95% CI 1.25‐4.67), security (aOR 2.56, 95% CI 1.68‐3.90), constitutionality and jurisprudence (aOR 2.40, 95% CI 1.51‐3.82), and partisan frames (aOR 3.62, 95% CI 2.45‐5.37) had a greater odds of including any policy mention. In contrast, emotion (aOR 0.58, 95% CI 0.46‐0.75) and firearm culture frames (aOR 0.28, 95% CI 0.13‐0.63) were associated with lower odds of any policy mention. The time period was not a significant predictor of policy mention.

### Subgroup Analysis: Health Frame Comparison

**Figure 5.**
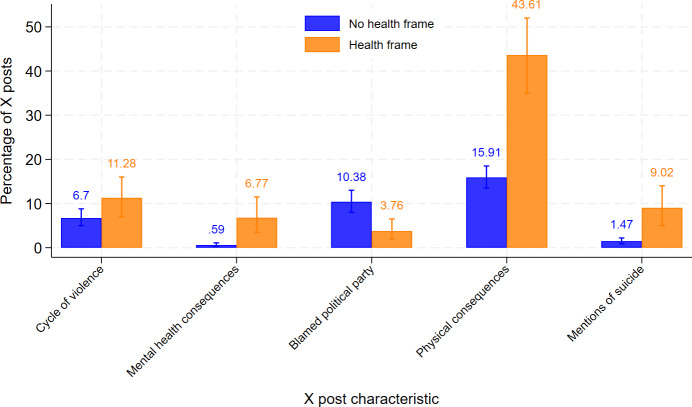
Descriptive unadjusted comparisons of significant characteristics by health frame usage in X posts by Pennsylvania state legislators (2017-2022).

Of the 1491 posts analyzed, 133 (9.4%) used health or public health frames. A greater percentage of posts that used health frames, compared to those that did not, mentioned suicide (120/133, 9% vs 20/1358, 1.5%; *P<*.001) and mental health consequences (9/133, 6.8% vs 8/1358, 0.6%; *P<*.001) (Example posts provided in Table 1). Physical consequences, such as mortality or disability, were referenced in 43.6% (58/133) of health-framed posts, compared to just 15.9% (216/1358) of posts without health framing (*P<*.001). Notably, causal blame directed toward a political party was less common in health-framed posts (5/133, 3.8%) than in those without health framing (141/1358, 10.4%; *P<*.001). Comparisons are illustrated in Figure 5.

## Discussion

### Principal Findings

This mixed methods analysis of X posts from Pennsylvania state legislators revealed key patterns in how firearm violence is framed and discussed in the political sphere. Legislators most frequently focused on mass shootings, often pairing them with symbolic expressions of remembrance and emotional appeals, and rarely connected these tragedies to specific policy proposals. Emotional frames, particularly those evoking moral outrage, were common, yet typically lacked actionable next steps. Health frames were rarely used. Of note, partisan rhetorical framing increased over the study period. Concurrently, causal blame was increasingly directed toward political parties and individual policymakers. Most posts relied on general advocacy rhetoric and nonspecific causal attributions that, in turn, yielded vague or generic policy suggestions. These patterns provide insight into the prevailing rhetorical strategies used by policymakers and underscore the opportunities for more evidence-informed communication, especially approaches grounded in public health, to more meaningfully address firearm violence.

Despite only accounting for 1%‐2% of firearm injuries, mass shootings dominate public discourse, overshadowing more prevalent forms of firearm violence, such as suicide [50]. Between 2019 and 2023 in Pennsylvania, 56% of firearm deaths were by suicide, 40% by homicide, and 1% by unintentional injury [51]. This is particularly salient for rural communities, where firearm suicide rates are high and health infrastructure is often limited [52]. While suicides account for the majority of firearm fatalities, most nonfatal firearm injuries result from interpersonal or community violence [53]. This may represent a gap in legislators’ knowledge or lack of acknowledgment of violence epidemiology and can undermine support for interventions targeted at the leading drivers of firearm injury [54-56].

Policymakers may also struggle with how to engage in ethical, nontrauma-inducing communication about suicide, contributing to a void in public rhetoric. This presents an opportunity for public health experts, communication specialists, and policymakers to cocreate trauma-informed messaging and reframe policy narratives around firearm suicide prevention, such as those provided by the American Foundation for Suicide Prevention, which is an approach that could foster broader support for evidence-informed injury prevention strategies [57]. In parallel, these same groups can collaborate to highlight the underlying structural drivers of community firearm violence, rather than focusing on event-specific details that risk retraumatization, to advance upstream, evidence-informed approaches to reduce both fatal and nonfatal firearm injuries [58].

Much of the current rhetoric by Pennsylvania legislators falls within a generalized advocacy frame characterized by emotional appeals in the aftermath of mass shootings. While these emotions are certainly valid and highlight the devastating toll of firearms, they are rarely accompanied by concrete calls to action or specific policy solutions. Instead, policymakers often rely on vague language, blaming “weak laws” or issuing nonspecific calls for change, without tying these claims to clear legislative proposals. This pattern, particularly when paired with highly emotional framing, may diminish the utility of such messages for constituents, colleagues, and coalition-building efforts. Policymakers may choose to remain nonspecific to appeal to a broad base of constituents with diverse political and ideological views in order to secure their electoral seat. For example, in a study of members of parliament (MPs) in the United Kingdom House of Commons, speeches by electorally vulnerable and junior MPs contained higher levels of emotive language compared to MPs with electoral security [59]. These emotions were mainly anger, disgust, fear, and sadness, which mirrors rhetoric used to characterize mass shootings. Recognizing that policymakers, like the broader public, process tragedy through emotion underscores the need to respond with empathy rather than condescension. A valuable next step is to explore how emotional responses can be intentionally channeled into public health communication that drives specific policy outcomes.

Policymakers may view social media as an ill-suited platform for nuanced policy discussion, even though prior research suggests it can influence policy agendas. Our data indicate that policymakers often use social media to emphasize partisan talking points rather than share information grounded in public health or scientific evidence. This is not surprising. As social media use has grown, platform algorithms have amplified divisive content, reinforcing users’ preexisting beliefs and deepening political polarization, including among policymakers themselves [60]. These dynamics unfold alongside growing mistrust in both political and public health institutions. In this context, and amid pressure to appeal to voter bases critical for nomination and electoral success, policymakers may feel compelled to conform to dominant partisan and polarized rhetoric, limiting their ability or willingness to communicate in ways that promote shared understanding or evidence-informed policy.

Despite broader political dynamics, current messaging techniques present an opportunity for refinement. For instance, when legislators directly attributed firearm violence to factors, such as firearm availability, accessibility, or ownership, they were more likely to propose concrete policy responses. This suggests that greater specificity in causal attribution can support more actionable policy discourse. A strengths-based approach, which builds on policymakers’ existing communication skills while encouraging improved clarity, accuracy, and alignment with public values, to message crafting could help policymakers pivot from general advocacy toward more effective communication strategies rooted in specificity, accuracy, and resonance with public values [61-64].

Although health-related frames were only used in 9.4% of legislators’ posts, they tended to appear in posts discussing the mental and physical consequences of firearm violence and were more likely to reference empirical evidence rather than partisan rhetoric. However, noticeably, health frames were used reactively to discuss the consequences of firearm violence rather than focus on firearm violence prevention, which is central to a public health approach. Health and public health frames have demonstrated success in advancing policy agendas, particularly when rooted in the authority and credibility of health care professionals [65]. Additionally, centering health garners favor from individuals across ideological spectrums for firearm injury prevention policies, such as extreme risk protection orders [66]. A study by Ojo et al [65] compared content, affect, and authorship of posts using the #ThisIsOurLane hashtag with #GunViolence over 1 year. Posts authored by health care providers and containing the #ThisIsOurLane hashtag were more likely to be framed through a health or public health-specific lens, connote positive emotions, and to contain more action-oriented content. This highlights the potential of health frames to amplify evidence-informed, preventive, and solutions-oriented messaging in the firearm policy space.

In addition to incorporating health frames, legislators might benefit from communication strategies that emphasize shared values, common goals, and inclusive identities, all of which can reduce polarization and foster greater message acceptance [60]. For example, framing firearm safety as a way to protect “Pennsylvanians,” families, children, and community well-being may appeal across ideological divides. Self-affirmation and trust-building techniques, such as acknowledging shared concerns and engaging constituents with empathy, may also help bridge divides. Similarly, emphasizing social norms (eg, “most parents support secure firearm storage”) and humanizing the communicator (eg, “as a parent and former law enforcement officer, I worry about...”) can increase message relatability and trust [60]. These strategies, shown to build common ground and reduce defensiveness, may help legislators communicate more effectively about contentious topics while still advancing specific, evidence-informed policy solutions. Further, resources exist to guide journalists in ethical, accurate, and equitable reporting on mass shootings and community firearm violence [58,67]. Although these guidelines are designed for journalists, their principles, when coupled with media literacy training, could inform the development of evidence-based communication guidelines for policymakers, who also play a key role in shaping public discourse and preventing harm.

Public health experts, clinicians, and clinician-researchers have a longstanding tradition of patient-centered communication and are well-positioned to collaborate with policymakers by translating complex evidence into accessible messages that minimize harm. Social media represents a natural extension of health professionals’ communication skills; however, few health professionals have training in public-facing media literacy or large-scale message framing. By integrating the expertise of health care professionals, the skills of communication specialists, and the platforms of policymakers, there is a clear opportunity to advance health-oriented framing and reshape firearm policy narratives to emphasize prevention as a form of health promotion [68,69]. For example, in the Agree to Agree campaign, health professionals partnered with the Ad Council, a communication firm, to develop public-facing messaging that helps caregivers discuss secure firearm storage with other caregivers [70]. Similarly, Da El Siguiente Paso (Take the Next Step), developed in collaboration with the Ad Council, offers a culturally relevant secure storage campaign for Hispanic firearm owners [71]. This bidirectional relationship also offers an opportunity to humanize both policymakers and health professionals, fostering trust and reducing stereotypes on both sides [60].

Building on this, future studies should broaden the scope of analysis by tracking engagement metrics from constituents and the general public, incorporating more states, and examining longer time frames. Additionally, similar to chart-stimulated recall used in health services research, involving policymakers in qualitative analysis of their own posts may foster reflective discourse that identifies barriers and facilitators to incorporating health framing and evidence into public communications. There is also considerable potential to leverage machine learning and large language models to support this work at scale. This descriptive analysis lays the groundwork for cocreating more accurate, ethical, and equitable portrayals of firearm violence on social media. Future research can further explore how health framing and survivor testimony influence message effectiveness and policy uptake, particularly the relationship between health-specific language and specific evidence-informed policy mentions.

Our findings highlight the potential importance of concerted collaboration between policymakers, advocates, researchers, and community partners to amplify survivor stories, honor lives lost, and promote specific, actionable steps for reform. Messages that combine health framing, evidence-informed policy, and emotional resonance may be especially effective in shifting public opinion and mobilizing support for firearm injury prevention. Enhancing the precision and relevance of firearm-related messaging, grounded in health and public health principles, can help bridge the gap between tragedy and legislative action. Providing policymakers and their staff with communication templates rooted in data and health equity may ultimately strengthen efforts to advance firearm violence prevention policies and promote individual and community well-being.

### Limitations

Our study has several limitations. First, our sample was limited to X posts and did not include other traditional or social media platforms that many policymakers also use to communicate. We also did not track user engagement metrics, such as reposts or likes, which could help assess the reach and resonance of specific messages. We opted to analyze the frames and solutions proposed in order to offer greater insight into policymaker perspectives rather than examining engagement metrics. Because we conducted a qualitative content analysis, incorporating multiple sources and studying engagement patterns was beyond the scope of this project. Second, the advocacy and endorsement frame was broadly coded to include general advocacy toward a cause or solution, specific policy advocacy, and self-advocacy. This broad categorization may have contributed to its strong correlation with policy mentions—both general and specific. The advocacy frame warrants more granular analysis and will be explored in future work. Third, assessing individual policymaker attributes or district characteristics was outside the scope of this study. However, future research could examine how district demographics and political context may influence rhetorical strategies. Fourth, regression results should be interpreted with caution due to the relatively small sample size. This limitation informed our decision to rely primarily on descriptive prevalence rather than model-driven inference. These associations should be interpreted as correlational rather than causal, due to the cross-sectional nature of the data. Fourth, our analysis did not account for external influences on social media rhetoric, such as concurrent events in the news cycle or geopolitical developments, which may have shaped the timing and content of posts. Fifth, although the study was not formally powered for inferential hypothesis testing, the sample was considered sufficiently large to ensure stability of model estimates and meaningful comparison between timepoints. Finally, our use of a priori frame definitions may have limited the capture of emergent frames; however, this approach ensured consistency and replicability across a large dataset and was balanced by iterative refinement during codebook development.

### Conclusion

By analyzing the language state legislators use when discussing firearm violence on social media, this study offered an empirical foundation to understand how political discourse aligns, or misaligns, with the health and public health realities. In describing firearm violence, Pennsylvania state legislators primarily focused on mass shootings, often relying on general advocacy rhetoric and vague causal attributions, which led to nonspecific policy proposals. This pattern persisted across all 3 time periods included in our analysis, even after adjusting for time using indicator variables in our regression models. These findings highlight opportunities for policymakers to leverage their strengths in communication, such as conveying emotion and personalizing complex issues, while becoming better informed about the epidemiology of firearm violence and evidence-based solutions. Adopting health-informed communication strategies can help advance specific policy recommendations to prevent all forms of firearm violence, especially firearm suicide.

## Supplementary material

10.2196/80397 Multimedia Appendix 1 Codebook used for qualitative content analysis.

## Abbreviations

MP: members of parliament
MSF: Multiple Streams Framework
REDCap: Research Electronic Data Capture
