# Supplementary material for: Firearm Violence and Health in Policymaker Discourse: Mixed Methods Social Media Analysis
Source: JMIR Form Res. 2025 Dec 16;9:e80397. doi: 10.2196/80397 (PMC12707438; doi:10.2196/80397)
Supplement: Multimedia Appendix 1 [file formative-v9-e80397-s001.docx]

**MULTIMEDIA APPENDIX**

**CODEBOOK**

| **Variable** | **Categories** | **Description/Notes** |
| --- | --- | --- |
| REDCap Record ID (Uneditable) | Integer | Unique REDCap Record ID Number |
| Quorum ID Number [DO NOT EDIT THIS FIELD] | Integer | Unique Quorum ID Number |
| Coder and/or Double Coder Name | Text | Identify Coder and/or Double Coder |
| X Post | Text | The Post will be populated into the field. |
| ***Domain 1: The Problem Stream*** | | |
| **General Content Analysis** | | |
| Does the post refer to a specific event or category of gun violence? | Binary Categorical (Y/N) | Refers to a specific firearm violence incident, compared to general discourse of firearms or firearm violence at large |
| Category of Shooting | 1. Mass shooting 2. Suicide or Intentional Self-Inflicted Harm 3. Unintentional or Accidental Injury 4. Interpersonal/Community Violence 5. Other | General Variable Definition: The post mentions or references a specific category of gun violence.   1. Mass Shooting: Naming of a specific Mass Shooting event. Also Defined by the FBI as a Shooting event that kills four people not including the perpetrator 2. Suicide or intentional self-inflicted harm: that is not caused by accidental injury 3. Unintentional or Accidental Injury: towards self or others 4. Interpersonal/Community: Refers to intentional firearm violence against another individual 5. Other: None of the above |
| Location of Violence | 1. School/School Grounds 2. Home 3. Place of Worship 4. Neighborhood/Community 5. Place of Business 6. City/County 7. Other Location | Location of shooting or general areas in which violence occurs if specified (ex. School shooting, school grounds, or near a school would all be considered a school shooting). Excluded “PA or Pennsylvania for an analysis.” Neighborhood/Community refers to a specific neighborhood/block/or geographic local within a city (ex West Philadelphia). City/County refers to the entire city or county(ies) mentioned. |
| Relationship of Perpetrator to Victims | 1. Intimate Partner 2. Parent/Guardian 3. Other Family Member 4. Law Enforcement 5. Other Authority Figure 6. Unknown to Victim 7. Other | 1. Specific references to violence towards a spouse, intimate partner, dating partner, or a person with whom someone shares a child. 2. Violence directed towards a child enacted by parent/guardian. 3. Other family member means cousin, uncle, aunt, sibling. 4. Law enforcement as perpetrator of violence 5. Other authority figure can include teacher, school personnel 6. X posts mentions that perpetrator Unknown to Victim 7. Other relationship |
| Mentions of Victim or Shooter | 1. Victim only 2. Shooter only 3. Victim and shooter 4. No specific mention | If victim is also the shooter (ex. in the case of self-directed injury), select “victim only” and ensure that the category of violence is specified in the variable options above. |
| **Expressions of Symbolic Reponses** | | |
| Expressions | 1. "Thoughts and Prayers" 2. Solidarity 3. Remembrance 4. None of the above | 1. Refers to specific mention of "thoughts" and/or "prayers" or similar emotional sentiments directed towards the victims or family member. 2. Referring to solidarity with a victim, family or community impacted by gun violence. 3. Refers to in memorium events such as during anniversaries of mass shooting |
| **Call to Action** |  |  |
| General Call to Action | Binary Categorical (Y/N) | A general call to action is an appeal to the reader to act without any specific instructions or actionable steps. For example, “it is time to act” or “we must put an end to gun violence.” The action in the latter post is “putting an end to gun violence.” Both of these examples are calls to support a general movement, however neither provide specific steps for the reader to achieve this. |
| Specific Call to Action | Binary Categorical (Y/N) | A specific “call to action” in media coverage is defined as any information that the public or policymakers can utilize to facilitate individual or collective action. It should answer the question “You the reader can act by doing/performing/supporting a certain action...” Examples of specific calls to action include passing legislation or voting. |
| Is the Specific Call to Action to Vote? | Binary Categorical (Y/N) | Does the post ask people to vote? This could be voting for elected officials OR voting for a specific piece of legislation, ballot measure, or amendment. |
| **Issue Framing:** Framing refers to the underlying structures of belief, perception, and meaning that shape how policy issues are understood. It influences how a problem is constructed, which in turn affects who becomes involved, what solutions are considered, and when opportunities for policy change may arise. Frames signal why an issue matters—shaping public perception and guiding attention toward particular values or concerns. They are conveyed through the tactics, language, and messaging strategies used to persuade or engage an audience. For examples, please refer to Table 1 in the main manuscript. | | |
| Morality | Binary Categorical (Y/N) | Any perspective — or policy objective or action (including proposed action) — that is compelled by moral doctrine or interpretation, logic, uty, honor, honesty/integrity, righteousness or any other sense of ethics or social responsibility. Calculated way for someone to think about right and wrong rather than emotion. Often, the phrase “common sense” use used to connote logic. |
| Advocacy/Endorsement | Binary Categorical (Y/N) | A way of presenting information that promotes a particular viewpoint, policy, or action. It is designed to persuade the audience to support a specific cause, agenda, or position. This type of frame often includes subjective elements such as endorsements, recognition of efforts, calls to action, and expressions of support or opposition.  This frame can be general or specific advocacy. For example, it can include advocating for general gun violence prevention (viewpoint) or protecting the rights of firearm owners through legislation (policy). It can also include advocating or promoting one’s own actions towards advancing a specific goal. A vote or sponsorship of a bill is an endorsement. |
| Emotional Appeal or Moral Outrage | Binary Categorical (Y/N) | Uses words and phrases to evoke sympathy, sorrow, and a sense of tragedy. Uses phrases to express injustice and moral outrage which can motivate audiences to act against an issue. Using facts or statistics to highlight the shear scope of an issue and evoke a sense of moral imperative. |
| Cultural Identity | Binary Categorical (Y/N) | Relates to social norms, trends, values, and customs. For example, it can include hunting and sporting events, shooting season in Pennsylvania. It may also refer to tradition and firearms as a heritage item. |
| Equality or Equity | Binary Categorical (Y/N) | Equality or inequality with which laws, punishment, rewards, and resources are applied or distributed among individuals or groups. Also, the balance between the rights or interests of one individual or group compared to another individual or group. |
| Criminology and Policing | Binary Categorical (Y/N) | Framing gun violence within the context of crime and law enforcement. Specifically, if the problem or solutions are grounded in crime prevention or law enforcement interventions (ex. longer sentences for perpetrators or criminal penalties for gun-related offenses); portrayals of gun violence that are intertwined with the criminal justice system (ex. highlighting gang violence, armed robberies); involvement of law enforcement as primary responders (as opposed to healthcare workers or community members). |
| Constitutionality and Jurisprudence | Binary Categorical (Y/N) | The constraints imposed on or freedoms granted to individuals, government, and corporations via the Constitution, Bill of Rights and other amendments, or judicial interpretation. This deals specifically with the authority of government to regulate, and the authority of individuals/corporations to act independently of government.  Examples: Civil libertarianism, citing encroachment on constitutional rights and individual liberties, most commonly the second amendment. Local laws/policies wrapped in legal or lawmaker language. Citation of legal cases are also included in this frame. |
| Safety/Defense/Protection | Binary Categorical (Y/N) | Safety, threats to security, and protection of one’s person, family, in-group, nation, etc. Generally, an action or a call to action that can be taken to protect the welfare of a person, group, nation sometimes from a not yet manifested threat. In this frame, the coder ask if the post is using rhetoric related to security/defense/protection to persuade you to take a stance or delineates specific steps to provide that security/defense/protection. For example, this frame could include lauding the use of a firearm/gun for self-protection or protecting the community, or protecting the community from gun violence itself. |
| Public Opinion | Binary Categorical (Y/N) | References to general social attitudes, polling and demographic information, as well as implied or actual consequences of diverging from or “getting ahead of” public opinion or polls. Often uses the phrases “most people support…” |
| Health and Public Health | Binary Categorical (Y/N) | Is the public health approach to curb gun violence as described by the Centers for Disease Control and Prevention (CDC) mentioned (must meet one criteria):  a. Does post cite statistics or the importance of research, generating data, risk factors, etc.?  b. Does the post mention using evidence-based practices or science?  c. Does the post mention evaluating the implementation of the methods used to prevent firearm violence? |
| Partisan | Binary Categorical (Y/N) | Defines the problems or solutions of gun violence through a partisan lens by touting one political party over another or castigating a specific political party. |
| Informational | Binary Categorical (Y/N) | The entire post should convey information which could include statistics, public health recommendations, information about an or event/campaign/vigil. While neutral on the surface, may not be felt as neutral since authors tend to use jarring statistics to drive home a point. There should be no partisan language in an post that is information-framed. |
| Religiosity or Spirituality | Binary Categorical (Y/N) | Relates to tying right to bear a firearm or firearm violence to religiosity or spirituality.  Ex. “God given right” or “may God bless this state” |
| ***Domain 2: The Policy Stream*** | | |
| **Causal blame or Causal Attribution: Persons, policies, or entities held responsible for firearm violence** | | |
| Mental health and/or Illness | Binary Categorical (Y/N) | Causal blame is attributed to instances where mental illness (depression, anxiety, bipolar disorder, psychosis) of an individual are causal blamed, OR lack of resources to address mental health and illness is causal blamed. |
| Weak Firearm Laws | Binary Categorical (Y/N) | Causal blame is attributed to instances where non-specific mentions of weak or lax policies have led to firearm violence. |
| Increased Ownership, Access, or Availability to Firearms | Binary Categorical (Y/N) | Causal blame is attributed to the increased ownership, access, or availability of firearms. This code specifically attributes causal blame to one of these three categories compared to the “weak firearm laws” variable. |
| Decreased Possession of Firearms | Binary Categorical (Y/N) | Causal blame is attributed to decreased access to firearms is causal blamed for worsening firearm violence. Colloquially corresponds to the phrase: “the only thing that stops a bad guy with a gun is a good guy with a gun.” |
| Sociopolitical and Structural Determinants of Violence and Health | Binary Categorical (Y/N) | Causal blame is attributed to structural inequities and sociopolitical determinants of health leading to disparate availability of physical, geographical, spatial, human, and financial resources, or the capacity of existing systems and resources. This includes lack of education, housing instability, poverty, lack of opportunity and employment, community disinvestment. |
| Stereotyped/Biased/Racist Beliefs | Binary Categorical (Y/N) | Causal blame is attributed to racist or stereotyped causal blame attributions towards one or more specific races, ethnicities, or religious identities. This may include stereotyped behaviors, cultures, attitudes, or practices. |
| Hatred/Hate Crime | Binary Categorical (Y/N) | Causal blame is attributed towards hate. This code must specify “hate” or “hate crime.” Co-select Stereotyped/Biased/Racist beliefs if a specific identifier for race, religion, ethnicity is included in this post. |
| Firearms in the Hands or Wrong People | Binary Categorical (Y/N) | Causal blame is attributed to firearms in the hands of the wrong person or people. This code includes causal blame attributed to isolated shooters, “lone wolf,” or radicalized shooter. This code refers to the colloquial phrase “guns don’t kill people, people kill people.” The person is to causal blame, the gun is not to causal blame. |
| Gang Violence/Organized Crime | Binary Categorical (Y/N) | Causal blame is attributed to specific gangs/sets/crews or organized crime directly involved in firearm violence. |
| Individual or Groups of Policymakers | Binary Categorical (Y/N) | Causal blame is attributed to policymaker(s) for their actions or inactions contributing to firearm violence. |
| Victim Blaming | Binary Categorical (Y/N) | Causal blame is attributed to a victim of firearm violence for their involvement in activities that exposed them to firearm violence. These could include involvement in criminal activities or substance use disorder. |
| Substance Use | Binary Categorical (Y/N) | Causal blame is attributed to substance use/and or involvement in the sale, distribution, or abuse of illicit substances. |
| Firearm Industry and Commercial Practices | Binary Categorical (Y/N) | Causal blame is attributed to the firearm industry as a commercial determinant of health – specifically around the enterprising and business of firearm manufacturing and sale. |
| U.S. Firearm Culture | Binary Categorical (Y/N) | Causal blame attributed to culturally held beliefs around firearms, often specific to the United States. |
| Firearm Lobby | Binary Categorical (Y/N) | Causal blame is attributed to the firearm lobby. For example, the influence of the National Rifle Association or Gun Owners of America. |
| A Specific Political Party or Affiliation | Binary Categorical (Y/N) | Direct or implied causal blame attributed to a particular political party for both action or inaction towards addressing or contributing to firearm violence. |
| Other Causal blame | Binary Categorical (Y/N) |  |
| No causal blame discussed/not applicable | Binary Categorical (Y/N) |  |
| **Consequences** | | |
| Economic/Financial | Binary Categorical (Y/N) | Relates to the costs, benefits, or monetary/financial implications of firearm violence. It can refer to the economic impact firearm violence has on an individual, family, community, or to the economy as a whole. |
| Physical Health | Binary Categorical (Y/N) | Relates to the impact on the physical health wellbeing of victims, survivors, families and communities impacted by firearm violence. References to mortality can be included in this code; however, this code should not be selected if there are mentions of specific numbers of victims/shooters. It must relate to mortality or physical disability as a direct consequence. |
| Mental Health or Illness | Binary Categorical (Y/N) | Relates to the impact on the mental health wellbeing of victims, survivors, families and communities impacted by firearm violence. The post must mention some specificity related to mental health including depression, anxiety, PTSD, oppositional defiant disorder, conduct disorder, ADHD, or trauma-response. |
| Cycles of Violence | Binary Categorical (Y/N) | Relates to the perpetuation of cycles of violence acknowledging and understanding of propagation of violence as a result of a violent event. |
| Secondary Trauma/Co-Victim | Binary Categorical (Y/N) | Relates to the secondary trauma or health impacts on family members, caregivers, friends, or community members of direct firearm violence survivors or victims. |
| Well-Being | Binary Categorical (Y/N) | Relates to the effects of a policy on individuals’ wealth, mobility, access to resources, happiness, social structures, ease of day-to-day routines, or quality of community life. |
| Other | Binary Categorical (Y/N) |  |
| **Policies Mentioned** | | |
| Generic or Nonspecific Policies | Binary Categorical (Y/N) | Uses vague language such as laws/programs/initiatives/reforms as solutions to firearm violence. Post does not provide a description of the policy entail. |
| Restrictive Firearm Ownership Policies | Binary Categorical (Y/N) | Refers to policies that restrict the ownership of firearms. |
| Permissive Firearm Ownership Policies | Binary Categorical (Y/N) | Refers to policies that permit the ownership of firearms. |
| Funding or support for Community-Based Violence Intervention Programs (CVIP)/Hospital-Based Violence Intervention Programs (HVIP) | Binary Categorical (Y/N) | Refers to both funding for programs, capacity building, implementation. |
| Funding or support for Firearm Research | Binary Categorical (Y/N) | Refers specifically to research. |
| Funding or policies that support Substance Use Disorder | Binary Categorical (Y/N) | Should refer to substance use disorder prevention and/or treatment. |
| Policies that hold the firearm industry accountable | Binary Categorical (Y/N) | For example, pursuing litigation against firearm manufacturers for unfair, unjust, or deceitful practices. |
| Increased funding for protective policies for law enforcement or the criminal justice system | Binary Categorical (Y/N) | Refers to increased policing or carceral policies. |
| Policies that address structural determinants of violence | Binary Categorical (Y/N) | Refers to structural determinants of violence such as lack of education, lack of secure housing, and poverty |
| Policies that hold the firearm lobby accountable | Binary Categorical (Y/N) | Refers to addressing misinformation or lack of evidence-based measures perpetuated by the firearm industry |
| Notes | Free text | Coder should include notes about post. Can refer to questions about the post, assistance with codes, etc. |
